# Immune-Checkpoint Inhibitors as the First Line Treatment of Advanced Non-Small Cell Lung Cancer: A Meta-Analysis of Randomized Controlled Trials

**DOI:** 10.7150/jca.34677

**Published:** 2019-10-17

**Authors:** Yuqiao Chen, Yuan Zhou, Lu Tang, Xiong Peng, Hong Jiang, Guo Wang, Wei Zhuang

**Affiliations:** 1 Department of Thoracic Surgery, Xiangya Hospital of Central South University, 410008 Changsha, Hunan, People's Republic of China.; 2 Department of Neurology, Xiangya Hospital, Central South University, 410008, Changsha, Hunan, People's Republic of China.; 3 Department of Clinical Pharmacology, Xiangya Hospital, Central South University, Changsha 410008, Hunan, People's Republic of China.

## Abstract

**Background**: This meta-analysis aimed to explore if immunotherapy or chemotherapy alone or in combination is a better first line treatment strategy for advanced non-small cell lung cancer (NSCLC) patients.

**Methods**: Electronic databases including Google Scholar, PMC, PubMed, EMBASE, Scopus and the major conference proceedings were searched for relevant randomized controlled trials (RCTs) comparing outcomes of immune-checkpoint inhibitor combined with chemotherapy or immune-checkpoint inhibitor alone over chemotherapy alone in patients with advanced NSCLC without previous treatment. Study heterogeneity was assessed using the I2 test.

**Results**: A total of 14 RCTs including 8,081 treatment naïve advanced NSCLC patients were enrolled in this study. Our results showed that in comparison to chemotherapy alone, introducing immunotherapy into first-line chemotherapy has significant benefit in tumor response (RR, 1.27; 95% CI, 1.09 to 1.48), progression-free survival (PFS) (HR, -0.43; 95% CI, -0.56 to -0.31), and overall survival (OS) (HR, -0.30; 95% CI, -0.45 to -0.14) but with an increased risk of grade3 - 5 toxicity (RR, 1.11; 95% CI, 1.04 to 1.18). The pooled results of comparison of immune therapy alone with chemotherapy alone in selected patients with positive expression of Programmed Death-ligament (PD-L1) or with a high tumor mutational burden, demonstrated similar tumor response (RR, 1.13; 95% CI, 0.88 to 1.46), 3 - 5 grade toxicity (RR, 0.69; 95% CI, 0.40 to 1.19) and long-term outcomes, including OS (HR, -0.20; 95% CI, -0.43 to 0.03) and PFS (HR, -0.24; 95% CI, -0.61 to 0.14).

**Conclusions**: Our meta-analysis showed the superiority of combination therapy over monotherapy with chemotherapeutic agents in terms of tumor response, and long-term survival, but with an increased the 3 - 5 grade toxicity. And immune-checkpoint inhibitors alone showed similar tumor response, toxicity and long-term outcomes compared to platinum-based chemotherapy in selected patients.

## Introduction

Lung cancer is one of the most lethal diseases and has become the leading cause of cancer related deaths 1, 2. Non-small cell lung cancer (NSCLC) is the largest subtype of lung cancer, comprising approximately 85% cases 3, 4. The first-line treatment strategy for advanced NSCLC is based on the expression of oncogenic aberrations, such as epidermal growth factor receptor gene (*EGFR*), anaplastic lymphoma kinase gene (*ALK*), and orphan receptor tyrosine kinase (*ROS*) 1, 5. However, most patients with NSCLC do not harbor these genetic aberrations; thus, cytotoxic chemotherapy is still the first-line treatment for such patients 6, 7. Chemotherapy alone is associated with a median overall survival (OS) of 8 - 10 months, progression-free survival (PFS) of 4 - 6 months, and objective tumor response rates of 25 - 35% 8. Moreover, the toxic effects of platinum-based chemotherapy are a concern for both clinicians and patients, as these severely impair quality of life 9. Therefore, developing new agents with better effectiveness and less toxicity is crucial.

Drugs interrupting immune checkpoints, such as anti-cytotoxic T-lymphocyte-associated protein 4 (CTLA-4), anti-programmed cell death protein 1 (PD-1), anti-programmed cell death-ligand 1 (PD-L1), and others, can enhance anti-tumor immunity and mediate durable cancer regressions 10, 11. Previous studies have demonstrated promising therapeutic value of immune checkpoint inhibitors as these lead to improved tumor responses, prolonged long-term survival, and less toxicity for patients with metastatic NSCLC who had progressed during or after platinum-based chemotherapy 11-16. However, patients with advanced NSCLC usually undergo rapid deterioration during the first course of treatment, and less than half of these patients receive second-line therapy 17.

Over time several studies exploring the safety and efficacy of immunotherapy as the first-line treatment strategy for advanced NSCLC have been published 18-32. Due to the availability of a wide range of immunotherapeutic agents and distinct treatment strategies, there is no unanimous conclusion about the therapeutic status of immunotherapy in the management of naïve NSCLC patients. Thus, we performed this meta-analysis of randomized controlled clinical trials (RCTs), which included patients with locally advanced NSCLC with metastasis, to ascertain whether immune checkpoint agents alone or in combination with chemotherapy improve survival outcomes in NSCLC patients who received chemotherapy alone as a first-line treatment.

## Material and methods

### Study selection

**Figure 1:**
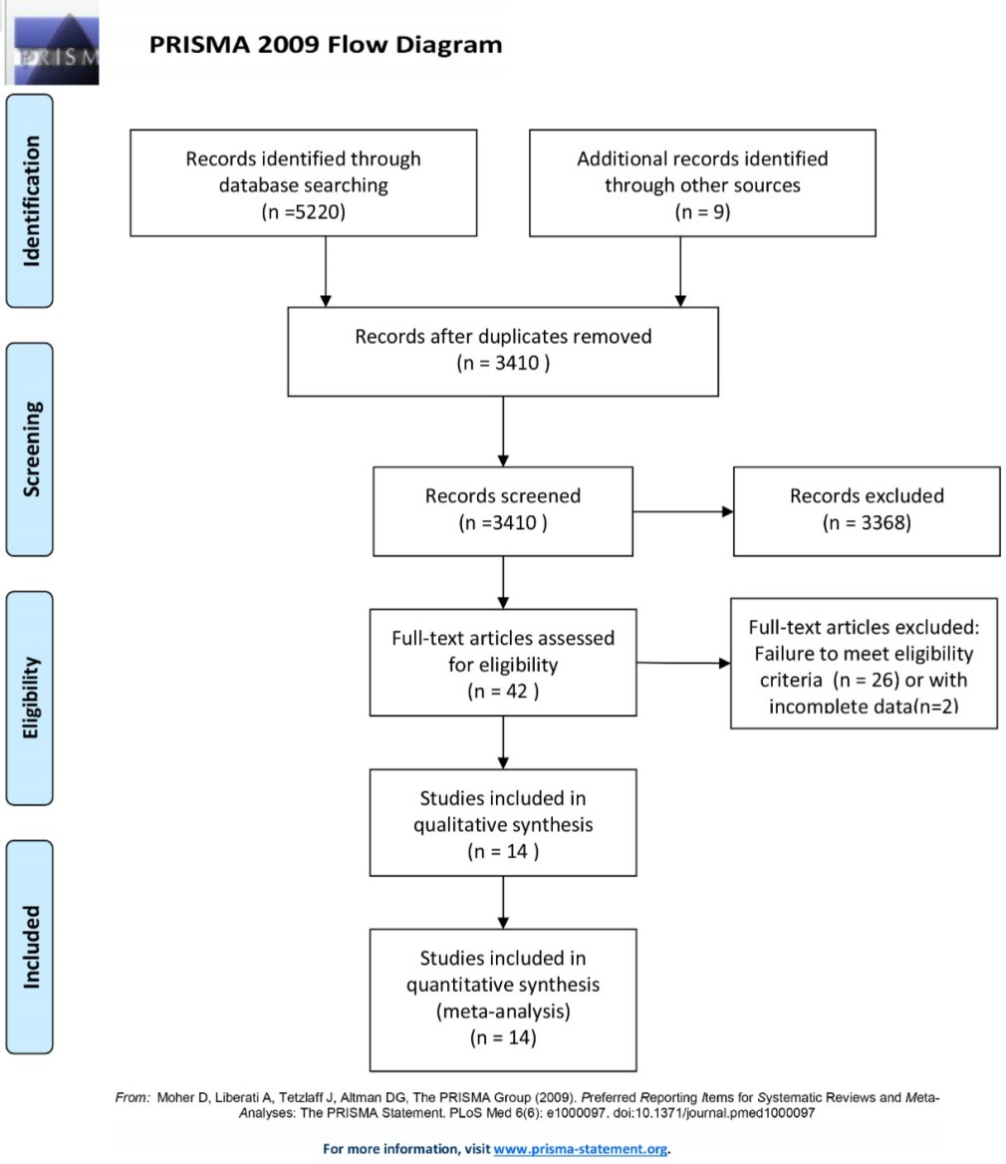
Flow chart depicting the selection algorithm and screening process.

Electronic databases including Google Scholar, PMC, PubMed, EMBASE, Scopus, and the major conference proceedings (the American Society of Clinical Oncology and the European Society for Medical Oncology) were searched by two authors (Chen and Zhou) independently for RCTs published between 1^st^ January, 2010 and 1^st^ June, 2019. The following medical subject heading (MeSH) terms were used: (1) "non-small cell lung cancer or NSCLC"; (2) "nivolumab or pembrolizumab or atezolizumab or ipilimumab or durvalumab"; (3) "PD-1 or PD-L1 or CTLA-4 or immune checkpoint inhibitor"; (4) "Randomized Controlled Trial or RCT". All potentially eligible and relevant clinical studies were manually retrieved and examined. Studies that met the following criteria were included in this meta-analysis: (a) RCTs; (b) studies comparing the combination of immune therapy and chemotherapy with chemotherapy alone in the treatment of advanced treatment-naive NSCLC patients; and (c) studies comparing immune therapy alone with chemotherapy alone in the treatment of advanced treatment-naive NSCLC patients. Non-randomized controlled trials, or studies unrelated to the first-line immune therapy, were excluded. Ultimately, 14 RCTs were included for quantitative analysis (Fig. 1). Any disagreements about the processes of study selection, data extraction, and methodological quality assessment were resolved by discussion and consensus with an independent expert (Zhuang).

### Data Extraction

The following information from the eligible studies were extracted by two authors (Tang and Chen) independently: year of publication, number of included patients, treatment regimen, and clinical outcomes. Clinical outcome measures included tumor response, long-term survival [progression-free survival (PFS) and overall survival (OS)], and the toxicity (3-5 grade toxicity and toxicity leading to discontinuation of treatment). Tumor response was stratified as objective responders who obtained a complete or partial response and as non-responders who experienced a stable or progressive disease according to the Response Evaluation Criteria in Solid Tumors (RECIST) version 1.1 33.

### Assessment of methodological quality

Two authors (Chen and Zhou) independently assessed the methodological quality of the eligible studies according to the Cochrane Collaboration guidelines v5.1.0 34.

### Data analysis

All statistical analyses were performed using Stata12.0 software (Stata Corporation, College Station, TX, USA). Risk ratios (RRs) and hazard ratios (HRs) with 95% confidence intervals (CIs) were calculated for dichotomous data. Heterogeneity among these included studies was evaluated using I^2^ statistics, where an I^2^ value > 50% was defined as substantial heterogeneity according to the Cochrane Collaboration guidelines v5.1.0 34. When I^2^ was < 50%, the fixed-effects model was used to assess outcomes; otherwise, the random-effects model were preferred. Sensitivity analysis using both fixed and random-effect models for the same data was conducted to confirm the robustness and reliability of the results.

## Results

### 1.1. Search strategy

The database search retrieved 5220 records. After deleting duplicate results, a total of 3410 abstracts were screened for eligibility, and 42 clinical trials were considered potentially eligible for inclusion based on titles and abstract review. After retrieving and further analyzing the full-text of these studies, another 28 studies were excluded; the remaining 14 studies 18-32 were finally included in the meta-analysis (Fig. 1). These 14 RCTs included a total of 8,081 patients: 4,391 patients had been administered immunotherapy alone or combination of chemotherapy and immunotherapy, and the remaining the 3,690 patients had been administered platinum-based chemotherapy alone. The characteristics of all 14 included studies are shown in Table 1. Among these 14 included studies, ten RCTs had compared combination of checkpoint inhibitor and chemotherapy with chemotherapy alone 18-26, 31, 32. The other four RCTs had compared checkpoint inhibitor alone with chemotherapy alone 27-30.

### 1.2. Outcome assessments

#### 1.2.1 Tumor response

**Figure 2:**
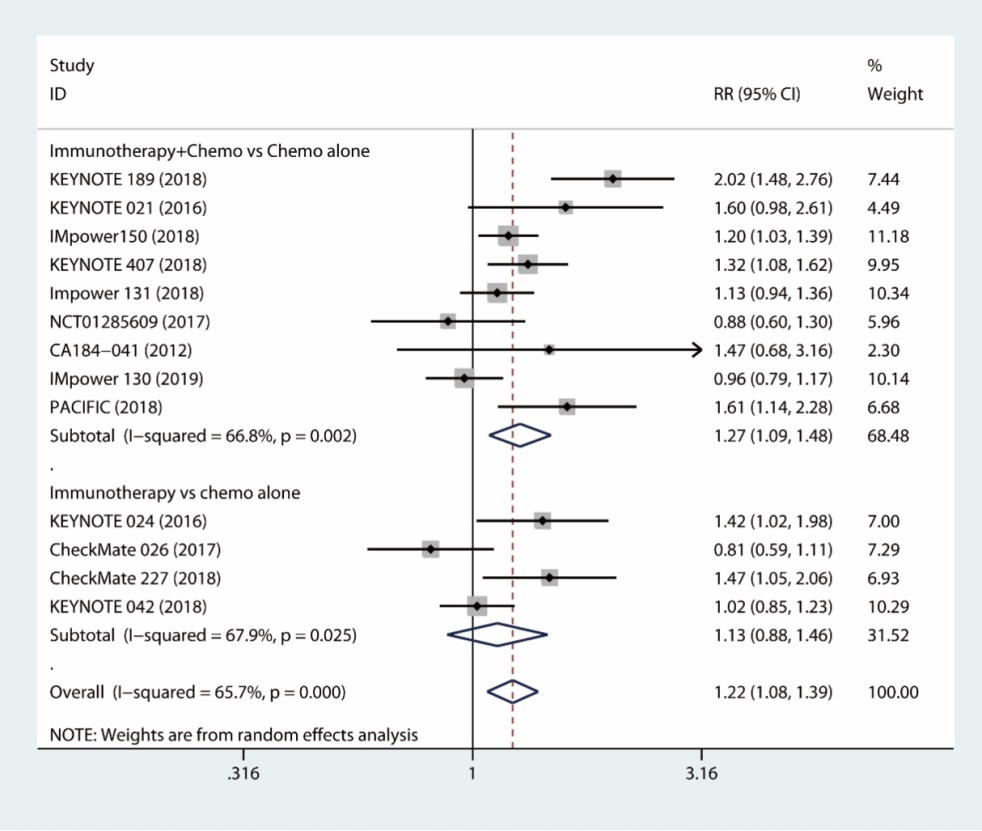
Forest plots of tumor response comparing combination therapy or immunotherapy alone versus chemotherapy alone.

The results based on ten RCTs 18-26, 31, 32 showed that combined immunotherapy and chemotherapy had significant benefit compared to chemotherapy alone with respect to tumor response (RR, 1.27; 95% CI, 1.09 to 1.48; I^2^ = 66.8%) (Fig. 2). Additionally, the immune-checkpoint inhibitor alone was not inferior to chemotherapy alone as the first-line therapy with respect to tumor response rate (RR, 1.13; 95% CI, 0.88 to 1.46; I^2^ = 67.9%) 27-30 (Fig. 2). As a whole, the use of the immunotherapy as the first-line therapy increased the objective tumor response (RR, 1.22; 95% CI, 1.08 to 1.39; I^2^ = 65.7%) (Fig. 2).

### 1.3. Toxicity

**Figure 3:**
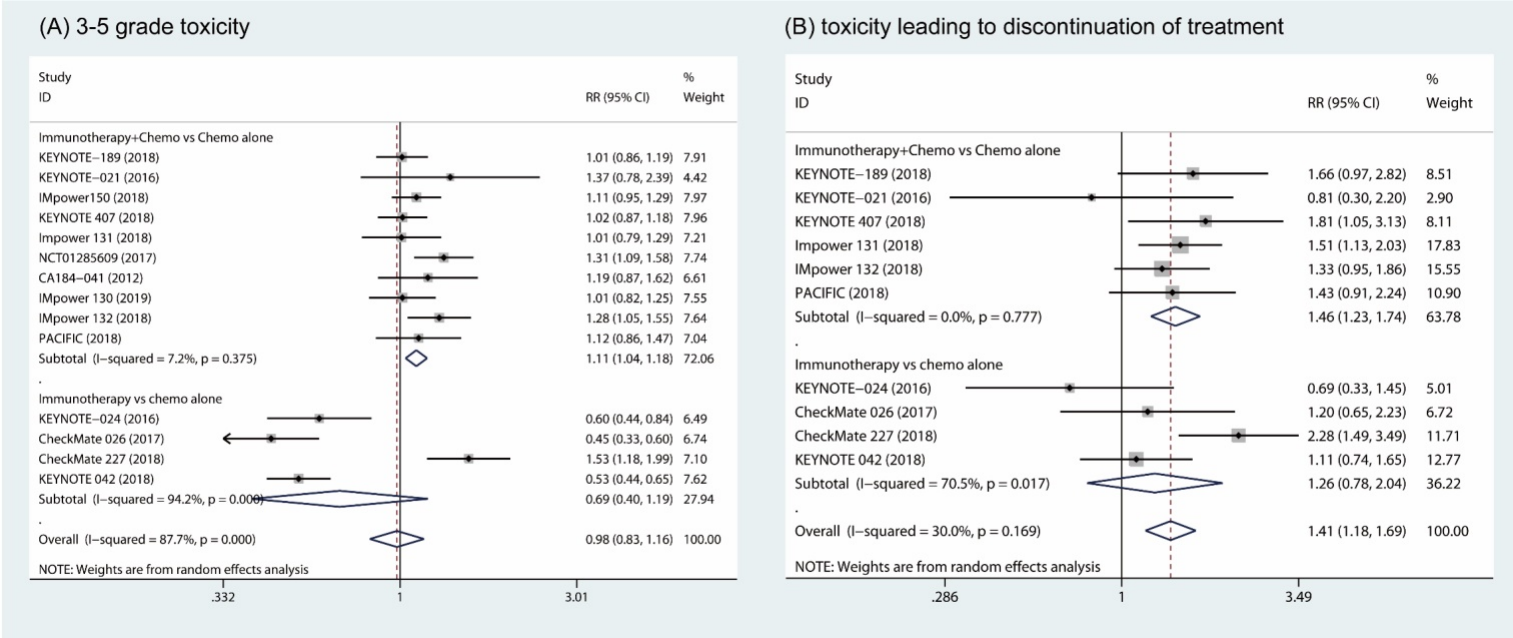
Forest plots of 3 - 5 grade toxicity comparing combination therapy or immunotherapy alone versus chemotherapy alone (A). Forest plots of toxicity leading to discontinue of treatment comparing combination therapy or immunotherapy alone versus chemotherapy alone (B).

The pooled results showed that the combination of immunotherapy and chemotherapy significantly increased toxicity compared to chemotherapy alone (RR, 1.11; 95% CI, 1.04 to 1.18; I^2^ = 7.2%) 18-26, 31, 32. However, no significant difference in 3 - 5 grade toxicity was found between patients in the monotherapy arms (RR, 0.69; 95% CI, 0.40 to 1.19; I^2^ = 94.2%) (Fig. 3A) 27-30. Furthermore, more patients who underwent the combination of immunotherapy and chemotherapy discontinued their treatment due to the toxicity in combination of immunotherapy and chemotherapy group compared to chemotherapy alone (RR, 1.46; 95% CI, 1.23 to 1.74; I^2^ = 0%) 18, 19, 21-23, 31, 32. However, patients who discontinued their treatment due to toxicity was comparable between groups of immune therapy alone and chemotherapy alone (RR, 1.26; 95% CI, 0.78 to 2.04; I^2^ = 70.5%) (Fig. 3B) 27-30.

### 1.4. Progression-free survival and overall survival

**Figure 4:**
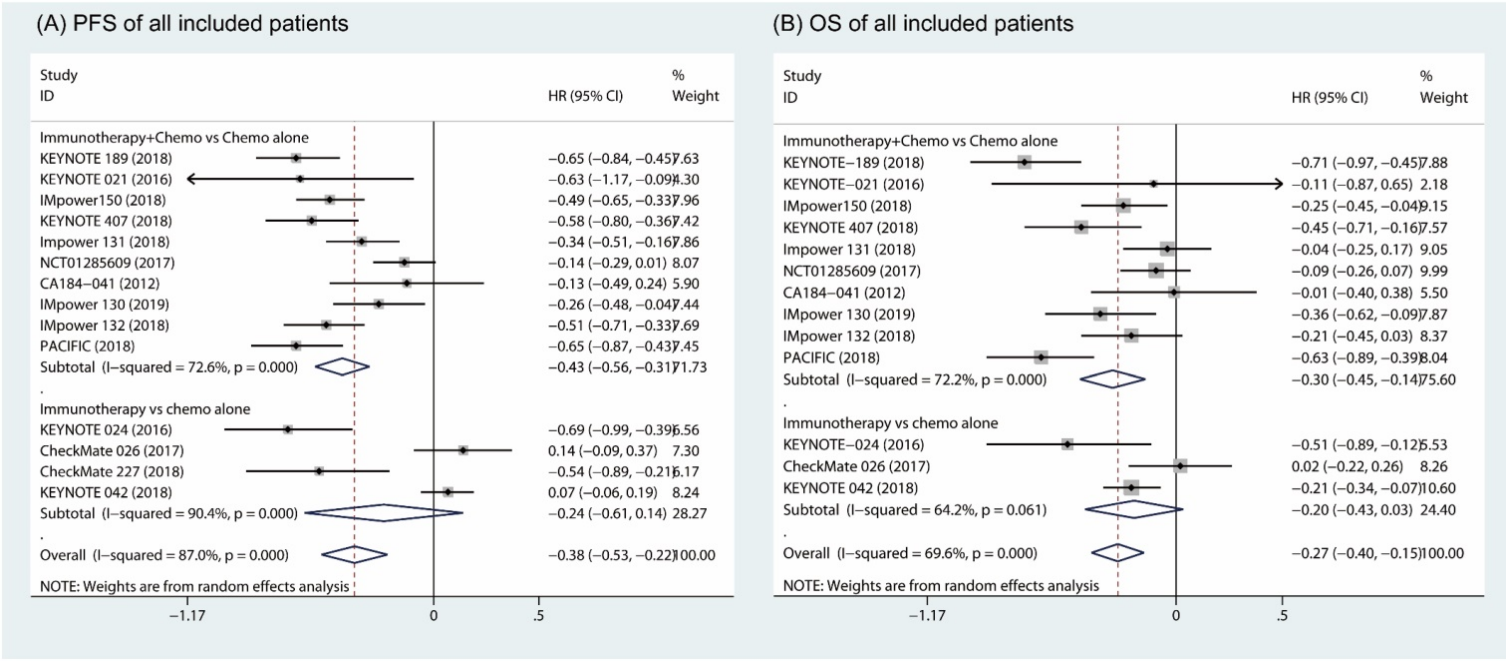
Forest plots of progress free survival comparing combination therapy or immunotherapy alone versus chemotherapy alone (A). Forest plots of overall survival comparing combination therapy or immunotherapy alone versus chemotherapy alone (B).

Based on random effects model analysis, a statistically significant benefit of combination of immune therapy and chemotherapy over chemotherapy alone was observed in term of PFS (HR, -0.43; 95% CI, -0.56 to -0.31; I^2^ = 72.6%) (Fig. 4A) 18-26, 31, 32. The OS also improved upon addition of an immune checkpoint inhibitor with chemotherapy as the first-line therapy (HR, -0.30; 95% CI, -0.45 to -0.14; I^2^ = 72.2%) (Fig. 4B). However, there was no significant difference between patients who received immunotherapy compared to those who took platinum-based chemotherapy in terms of PFS (HR, -0.24; 95% CI, -0.61 to 0.14, I^2^ = 90.4%; Fig. 4A) and OS (HR, -0.20; 95% CI, -0.43 to 0.03; I^2^ = 64.2%; Fig. 4B) 27-30.

**Figure 5:**
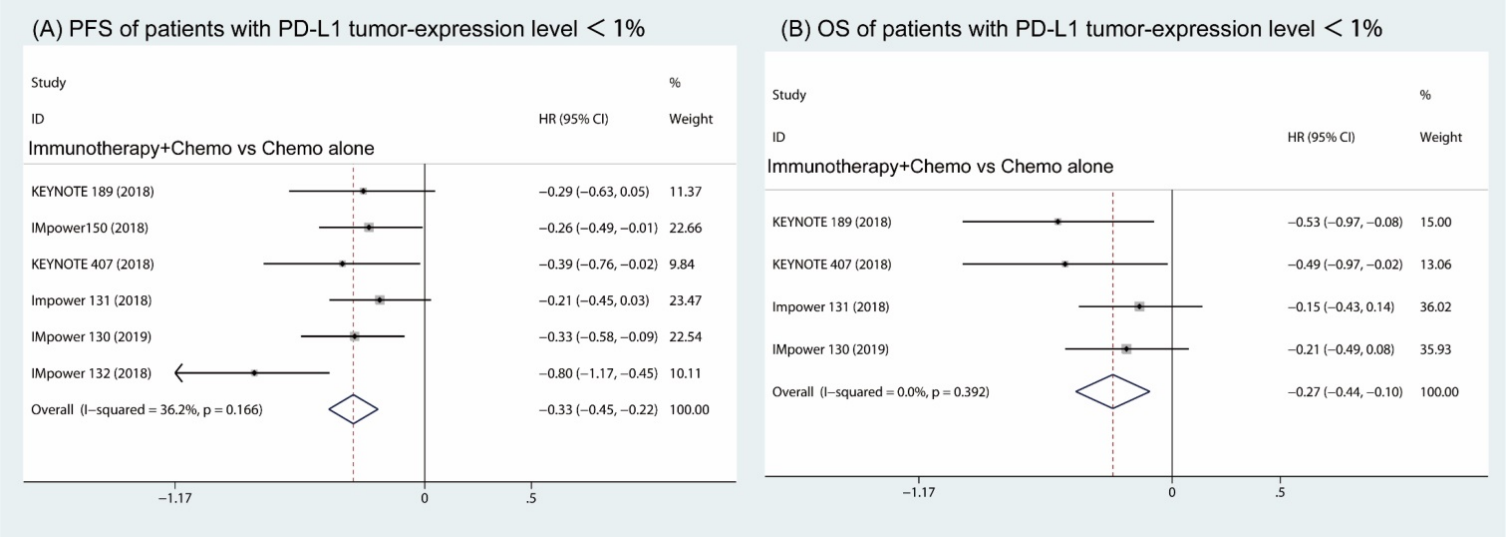
Subgroup analysis of patients with PD-L1 tumor-expression level < 1% in combination therapy versus chemotherapy alone. Forest plots of progress free survival (A) and overall survival (B).

Furthermore, the subgroup analysis of patients with PD-L1 expression less than 1% revealed that both the PFS and OS were prolonged in the combination of immune therapy and chemotherapy compared with chemotherapy alone (HR, -0.33; 95% CI, -0.45 to -0.22; I^2^ = 36.2% (Fig 5A) and HR, -0.27; 95% CI, -0.44 to -0.10; I^2^ = 0.0% (Fig 5B)).

### 1.5. Methodological quality and sensitivity analyses

**Figure 6:**
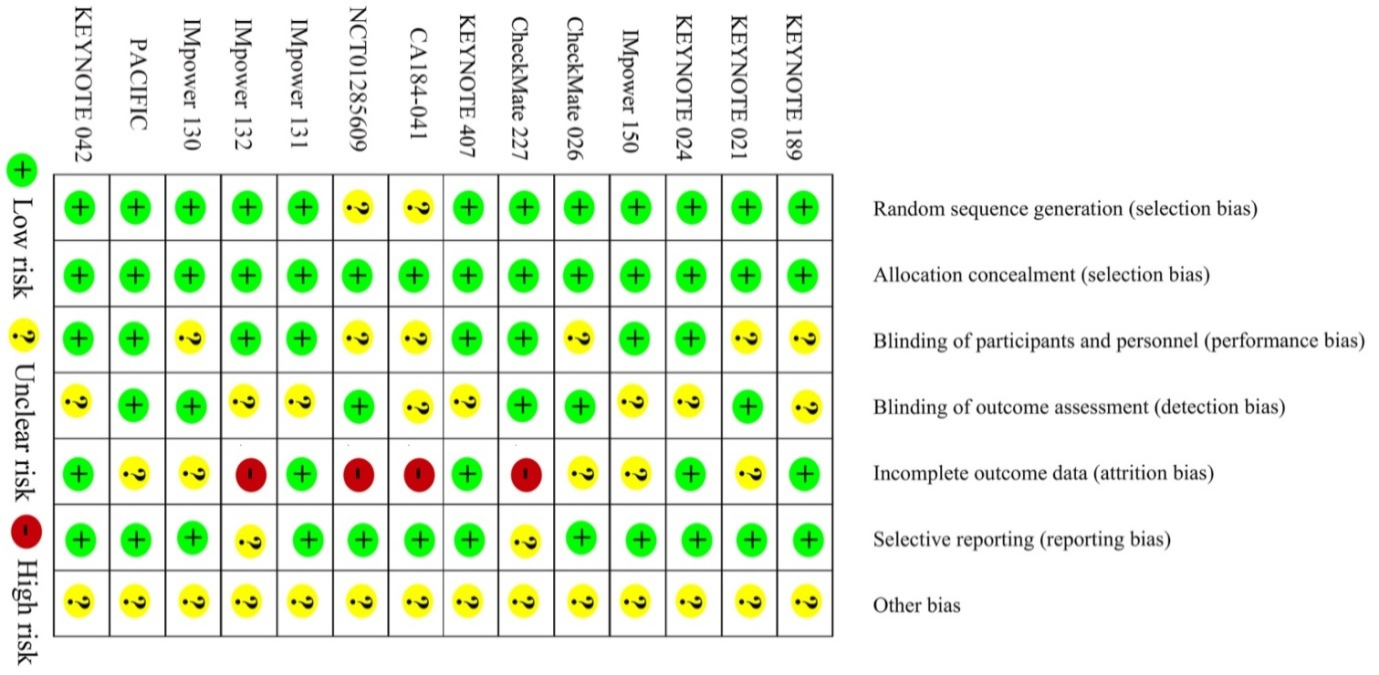
Risk of bias of the included trials.

The methodological quality of the eligible studies is shown in Figure 6. All studies were assessed as level A. The sensitivity analyses showed robustness and reliability of our results.

## Discussion

Our meta-analysis aimed to compare the treatment regimes of immunotherapy alone or in combination with chemotherapy with chemotherapy alone in patients with advanced treatment-naive NSCLC.

Based on all the available information extracted from the included trials, we found that combination of immunotherapy and platinum-based chemotherapy as a first-line therapy has a favorable long-term effect. It is important to note that the studies, which compared the combination of chemotherapy and immunotherapy with chemotherapy alone, included all treatment-naive patients regardless of PD-L1 expression on tumor cells. Due to the functional mechanism of the immune checkpoint inhibitor, expression levels of PD-L1 on tumor cells assessed by immunohistochemistry have been regarded as a potential responsive biomarker to these agents 14, 35. The previous study showed that the treatment efficiency in patients with a higher PD-L1 tumor-expression level was significantly better than those with a lower PD-L1 tumor-expression level, when treated by the immune checkpoints inhibitor 36, 37. But, the subgroup analysis of this study showed that patients who had low or negative expression of PD-L1(≤1%) also benefitted from combined immunotherapy and chemotherapy as a first-line treatment in both PFS and OS. Although favorable long-term survival was observed in combined immunotherapy and chemotherapy group, the initial 3-6 month survival curve often overlaps or even crosses 38, which means that the efficacy of immunotherapy combined with chemotherapy in the early stage of treatment may not be superior to chemotherapy^18^-^26^. The higher rate of toxicity might offset the therapeutic effect of the combination of immunotherapy and chemotherapy at the onset of treatment 38.

Comparison of monotherapy arms of immune-checkpoint inhibitors and chemotherapy shows that selected patients with positive PD-L1 tumors 27, 29, 30 or a high mutation burden 28, who were administered immunotherapy alone, experienced longer PFS and OS. However, the difference was not significant with high heterogeneity among the studies. The results of the KEYNOTE-024 study demonstrated an advantage of pembrolizumab over chemotherapy in patients with a PD-L1 tumor-expression level ≥50% with regards to long-term survival. PFS improvement of 4.3 months was observed in the pembrolizumab group, despite the finding that 43% of patients who had undergone chemotherapy crossed over to the pembrolizumab group 29. The results of KEYNOTE-042 30 which recruited treatment-naïve stage IIIB-IV NSCLC patients with a PD-L1 tumor-expression level ≥1% demonstrated patients in the pembrolizumab group enduring a prolonged OS compared with chemotherapy group. The subgroup analysis of patients with a PD-L1 tumor-expression level ≥ 50% showed that the PFS and OS were significantly longer in pembrolizumab group compared with chemotherapy group. However, in patients with a PD-L1 tumor-expression level <50%, there was no significant difference in PFS and OS between the two groups 30. Based on these observations, we proposed that pembrolizumab alone is effective in patients a PD-L1 tumor-expression level ≥50%. However, in the CheckMate 026 study, no significant advantage of nivolumab over chemotherapy was observed in terms of objective tumor response and long-term survival among patients with PD-L1 expression >5% 27. The subgroup analysis demonstrated that even in patients with PD-L1 expression >50%, there is no significant difference between nivolumab monotherapy group and chemotherapy group. Positive detection of PD-L1 on tumor cells alone may not be sufficient to predict outcomes among patients who receive immunotherapy. Owing to the complexity of the immune system, patients with low- PD-L-1 expression may also benefit from immunotherapy. Thus, new biomarkers, such as tumor mutational burden for response to immune-oncological agent, beyond PD-L1 expression levels, may be the most critical markers for selecting patients for immunotherapy. The CheckMate 026 trial demonstrated that among patients with a high tumor-mutation burden, nivolumab monotherapy achieved a higher response rate compared to chemotherapy alone (47% *vs*. 28%). The median PFS of patients receiving nivolumab monotherapy (9.7 months) has been reported to be much longer than those receiving chemotherapy alone (5.8 months) 27. Furthermore, the CheckMate 227 trial demonstrated that PFS was significantly longer with first-line nivolumab plus ipilimumab (7.2 months) compared to chemotherapy (5.5 months) among NSCLC patients with a high tumor mutational burden (≥10 mutations per megabase), irrespective of PD-L1 expression levels.

Numerous limitations of these trials may hinder the fairness of this meta-analysis. Since only 14 RCTs were included in this meta-analysis, the results were underpowered. Furthermore, the HRs and corresponding 95% CIs were mainly extracted from the original studies without access to individualized data, which might have contributed to reporting bias. High heterogeneity was observed among the included studies, which may have decreased the strength of our meta-analysis. The high heterogeneity may be explained by the following: First, different immune checkpoint inhibitor regimens were used in different studies. Second, inclusion criteria differed among the included studies. Third, different treatment strategies had been used among studies.

The results of this meta-analysis demonstrated the superiority of combined immunotherapy with chemotherapy over chemotherapy alone in terms of tumor response and long-term survival as a first-line treatment strategy for advanced NSCLC patients but with higher rate of 3 - 5 grade toxicity. And immune-checkpoint inhibitors alone showed similar tumor response, toxicity and long-term outcomes compared to platinum-based chemotherapy in selected patients.

## Figures and Tables

**Table 1:** Summary of 14 randomized controlled trials included in the meta-analysis.

Study	Author	Year	Study Group (regime and no. of Pts.)		Control Group (regime and no. of Pts.)		Inclusion criteria
CA184-041	Lynch et al. 25	2012	Ipi+Chemo	70	Chemo alone	66	Stage IIIB or IV NSCLC
KEYNOTE 021	Langer et al. 19	2016	Pembro+Chemo	60	Chemo alone	63	Stage IIIB or IV, non-squamous NSCLC without targetable genetic aberration
NCT01285609	Govindan et al. 26	2017	Ipi+Chemo	388	Chemo alone	361	stage IV or recurrent squamous NSCLC
IMpower 150	Socinski et al. 20	2018	Atezo+Chemo	400	Chemo alone	400	Stage IIIB or IV, non-squamous NSCLC without targetable genetic aberration
IMpower 131	Jotte et al. 22	2018	Atezo+Chemo	343	Chemo alone	340	Stage IV, squamous NSCLC without
IMpower 132	Papadimitrakopoulou et al. 23	2018	Atezo+Chemo	292	Chemo alone	286	Stage IV non-squamous NSCLC without targetable genetic aberration
KEYNOTE 407	Paz-Ares et al. 21	2018	Pembro+Chemo	278	Chemo alone	281	Stage IV, squamous NSCLC
KEYNOTE 189	Gandhi et al. 18	2018	Pembro+Chemo	410	Chemo alone	206	Stage IV non-squamous NSCLC without targetable genetic aberration
IMpower 130	West et al. 24	2019	Atezo+Chemo	473	Chemo alone	232	Stage IV, non-squamous NSCLC without targetable genetic aberration
PACIFIC	Antonia et al. 31, 32	2018	Durva+Chemo	476	Chemo alone	237	stage III, unresectable NSCLC
KEYNOTE 024	Reck et al. 29	2016	Pembro alone	154	Chemo alone	151	Stage IIIB or IV, NSCLC without targetable genetic aberration but with a PD-L1 tumor-expression level of 50% or more
CheckMate 026	Carbone et al. 27	2017	Nivo alone	271	Chemo alone	270	Stage IV or recurrent NSCLC without targetable genetic aberration but with a PD-L1 tumor-expression level of 5% or more
CheckMate 227	Hellmann et al. 28	2018	Nivo+Ipi	139	Chemo alone	160	Stage IV or recurrent NSCLC without targetable genetic aberration, with a high tumor mutational burden (≥10 mutations per megabase)
KEYNOTE 042	Mok et al. 30	2018	Pembro alone	637	Chemo alone	637	Stage IV or recurrent NSCLC without targetable genetic aberration but with a PD-L1 tumor-expression level of 1% or more

**Abbreviations:** *Pts* -patients, *Pembro-* Pembrolizumab, *Atezo* -Atezolizumab, *Nivo-* Nivolumab, *Ipi-* Ipilimumab, *Durva*-Durvalumab, *Chemo-* Chemotherapy
